# Light-activated BioID – an optically activated proximity labeling system to study protein–protein interactions

**DOI:** 10.1242/jcs.261430

**Published:** 2023-10-11

**Authors:** Omer Shafraz, Carolyn Marie Orduno Davis, Sanjeevi Sivasankar

**Affiliations:** Department of Biomedical Engineering, University of California, Davis, Davis, CA 95616, USA

**Keywords:** E-cadherin, TurboID, Membrane protein, Optogenetic, Proximity labeling, LAB

## Abstract

Proximity labeling with genetically encoded enzymes is widely used to study protein–protein interactions in cells. However, the accuracy of proximity labeling is limited by a lack of control over the enzymatic labeling process. Here, we present a light-activated proximity labeling technology for mapping protein–protein interactions at the cell membrane with high accuracy and precision. Our technology, called light-activated BioID (LAB), fuses the two halves of the split-TurboID proximity labeling enzyme to the photodimeric proteins CRY2 and CIB1. We demonstrate, in multiple cell lines, that upon illumination with blue light, CRY2 and CIB1 dimerize, reconstitute split-TurboID and initiate biotinylation. Turning off the light leads to the dissociation of CRY2 and CIB1 and halts biotinylation. We benchmark LAB against the widely used TurboID proximity labeling method by measuring the proteome of E-cadherin, an essential cell–cell adhesion protein. We show that LAB can map E-cadherin-binding partners with higher accuracy and significantly fewer false positives than TurboID.

## INTRODUCTION

Protein–protein interactions (PPIs) are essential for cellular function, and decoding these interactions is crucial for understanding biological pathways in health and disease. Although many methods have been developed to map protein interactomes (Qin et al., 2021), proximity labeling (PL) techniques are among the most widely used approaches, given that they capture transient, dynamic PPIs in the near-native cellular environment.

PL employs enzymes such as promiscuous biotin ligases for proximity-dependent biotin identification (BioID) (Choi-Rhee et al., 2004; Roux et al., 2012; Kim et al., 2016; Ramanathan et al., 2018), peroxidases for peroxide-dependent biotin identification (APEX) (Martell et al., 2012), horseradish peroxidase for enzyme-mediated activation of radical source (EMARS) (Kotani et al., 2008) or Pup ligase PafA in PUP-IT (Liu et al., 2018). These enzymes are genetically fused to a protein of interest (the bait) and generate short-lived intermediate reactive biotins that covalently tag neighboring proteins (the prey). Other methods of tagging include using photosensitizers, such as the enzyme miniSOG (Zhai et al., 2022) or photocatalyst–antibody conjugates to generate singlet oxygen (Müller et al., 2021) or activated carbenes (Geri et al., 2020) as alternate intermediates for targeting biotin-tagged probes. Subsequently, biotin-tagged prey proteins are enriched using streptavidin-coated beads and identified using mass spectrometry (MS).

BioID is preferred over other PL methods in many *in vitro* and *in vivo* applications because peroxidases, such as APEX and HRP, require the addition of hydrogen peroxide and miniSOG produces reactive oxygen species, which can both be toxic to cells. However, BioID has long labeling times (>18 h) and requires an optimal temperature of 37°C (Roux et al., 2012). In an effort to improve the performance of BioID, TurboID has been introduced as a fast-labeling biotin ligase (10 min labeling time) that also retains activity at temperatures <37°C, which enables it to be used in organisms like flies, worms, yeast (Branon et al., 2018) and plants (Mair et al., 2019), as well as in cell culture (Shafraz et al., 2020). However, TurboID can biotinylate proteins using endogenous cellular biotin, which creates a large labeling background as well as cellular toxicity. To overcome this limitation, a ‘split-TurboID’ technology was recently introduced, which splits the TurboID enzyme into two inactive halves that only induce biotinylation when reconstituted by the addition of the chemical cofactor rapamycin. Although this reduces background labeling and increases spatial specificity (Cho et al., 2020a), the requirement that both halves of Split-TurboID be expressed in close proximity creates a level of background biotinylation unrelated to the addition of the cofactor (Cho et al., 2020b). Additionally, usage of a cofactor introduces the possibility of uncontrolled effects on cellular processes, and the time resolution is limited by the ability of the cofactor to diffuse within the cell. Furthermore, the difficulty of removing the factors from the medium makes it difficult to control the cessation of the biotinylation reaction (Klewer and Wu, 2019).

These limitations in conventional proximity labeling can be overcome by developing new classes of proximity labeling technologies that can be activated on demand without the addition of chemical cofactors, and with high spatial and temporal control. We therefore designed a proximity labeling technology that is precisely triggered using blue (488 nm) light. Our protein construct, called light-activated BioID (LAB), is generated by fusing the two halves of the split-TurboID enzyme to the genetically encoded *Arabidopsis thaliana* photodimeric proteins cryptochrome 2 (CRY2) and cryptochrome-interacting basic-helix-loop-helix (CIB1) (Liu et al., 2008). Upon exposure to blue light, CRY2 and CIB1 dimerize within 300 ms (Kennedy et al., 2010; Taslimi et al., 2016). The rapid dimerization of CRY2 and CIB1 reconstitutes the two halves of the split-TurboID enzyme and induces biotinylation. Given that the photodimers have a half-life of ∼6 min (Kennedy et al., 2010; Taslimi et al., 2016), dissociation of CRY2 and CIB1 re-splits the TurboID and halts biotinylation. Because the LAB dimer dissociates within minutes after the blue light is turned off, background biotinylation is reduced and false positives as well as cellular toxicity due to over-biotinylation are minimized.

Here, we benchmark LAB in different cell lines using live-cell imaging, immunofluorescence, western blots and MS. First, as a proof of concept, we target LAB to the cell membrane in human embryonic kidney (HEK) 293T cells and show that LAB dimerizes and biotinylates proximal proteins when exposed to blue light. Next, we validate LAB against stand-alone TurboID by measuring the proteome of the ubiquitous transmembrane cell–cell adhesion protein E-cadherin (Ecad; also known as CDH1) in Madin–Darby canine kidney (MDCK) cells. We demonstrate that LAB can map known Ecad-binding partners in a light-dependent manner, with higher accuracy and significantly fewer false positives compared to what is seen with stand-alone TurboID.

## RESULTS

### LAB dimerizes upon blue light illumination

**Fig. 1.**
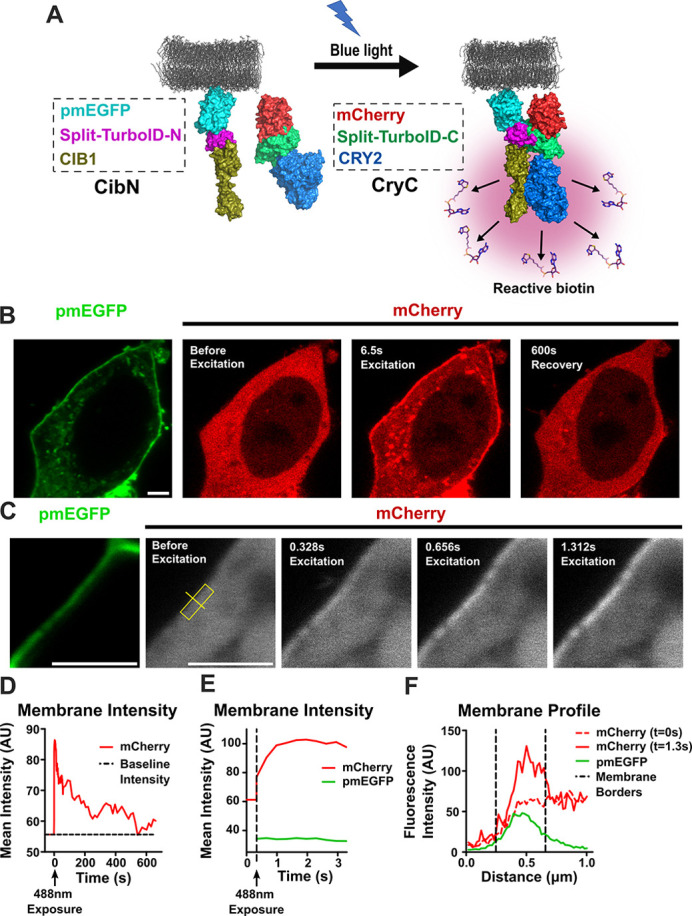
**LAB dimerizes upon blue light illumination.** (A) Schematic of LAB construct used in HEK293T cells. A fragment of Split-TurboID (N) and *Arabidopsis* protein CIB1 were fused onto prenylated plasma membrane targeted enhanced green fluorescent protein (pmEGFP), which localizes to the plasma membrane (CibN). The complementary Split-TurboID (-C) fragment was fused to blue-light receptor protein cryptochrome 2 (CRY2) and mCherry fluorescent protein (CryC) and expressed in the cytoplasm. When exposed to blue light, CRY2 interacts with CIB1 and reconstitutes the split-TurboID, which generates reactive biotin for proximity labeling. Both constructs were transiently co-expressed in HEK293T cells. (B) Confocal images showing CibN localizing to the membrane and CryC in the cytoplasm of the cells. When excited with a 488 nm laser, CryC localized to the membrane in seconds and dissociated completely in about 600 s. Scale bar: 3 µm. (C) A region of the membrane was excited with 488 nm laser and CryC accumulation was monitored. CryC accumulated at the membrane region in sub-seconds. Scale bars: 3 µm. (D) CryC intensity was measured on the membrane in B over a period of 10 min after a single 488 nm pulse exposure at *t*=0 min. The horizontal dashed line represents the baseline intensity measured pre-exposure. The intensity measurements show membrane CryC returning to baseline levels over a period of 10 min. (E) CryC and CibN intensity was measured across the yellow line in C with vertical black dashed lines delineating the membrane on the graph. The fluorescence intensity measurements showed a strong correlation between CryC and CibN after light exposure. (F) Integrated CryC (red) and CibN (green) intensity was measured in the yellow box in C over time, with the vertical black dashed line indicating 488 nm laser excitation. CryC fluorescence reached the maximum intensity within seconds, whereas CibN fluorescence remained constant. Data in B–F representative of three repeats. AU, arbitrary units.

We developed genetically encodable LAB constructs by fusing previously reported low-affinity split-TurboID (L73/G74) (Cho et al., 2020a) onto light-inducible dimerizing plant proteins CRY2 and CIB1. The N-terminal fragment of split-TurboID (spTN) and CIB1 were fused onto plasma membrane-targeted enhanced green fluorescent protein (pmEGFP). The C-terminal split TurboID fragment (spTC) and CRY2 were fused onto mCherry and expressed in the cytoplasm (Fig. 1A). We hypothesized that, upon blue light exposure, CRY2-spTC–mCherry (henceforth referred to as *CryC*) would translocate to the plasma membrane and bind to CIB1-spTN–pmEGFP (henceforth referred to as *CibN*) (Fig. 1A).

We transiently expressed both constructs in HEK293T cells and observed that CryC was distributed throughout the cytoplasm whereas CibN was localized at the plasma membrane (Fig. 1B). When illuminated with a 488 nm laser, CryC translocated to the plasma membrane within seconds of laser exposure; the residual mCherry signal in the cytoplasm even after laser exposure arose because CryC was expressed at high enough levels to saturate CibN on the membrane (Fig. 1B). When the laser beam was turned off, CryC dissociated back to the cytoplasm in ∼10 mins (Fig. 1B,D). These kinetics show that the fused constructs do not greatly alter previously reported CRY2 and CIB1 kinetics (Kennedy et al., 2010). Furthermore, when a selected region of the plasma membrane was illuminated with a 488 nm laser, CryC began accumulating in under a second and reached maximum accumulation in seconds (Fig. 1C,E,F). This demonstrates that LAB can be activated in selected regions of the cell and split-TurboID will be bound for ∼10 mins from the end of illumination, biotinylating neighboring proteins and giving more temporal and spatial control over previous PL systems. Although the interaction kinetics of our construct may differ slightly from previously reported values for CRY2 and CIB1 due to the use of fusion proteins, our data suggest that CryC and CibN associate and dissociate at rates largely similar to those published in previous reports (Kennedy et al., 2010; Taslimi et al., 2016).

### LAB biotinylates only in the presence of blue light and biotin

**Fig. 2.**
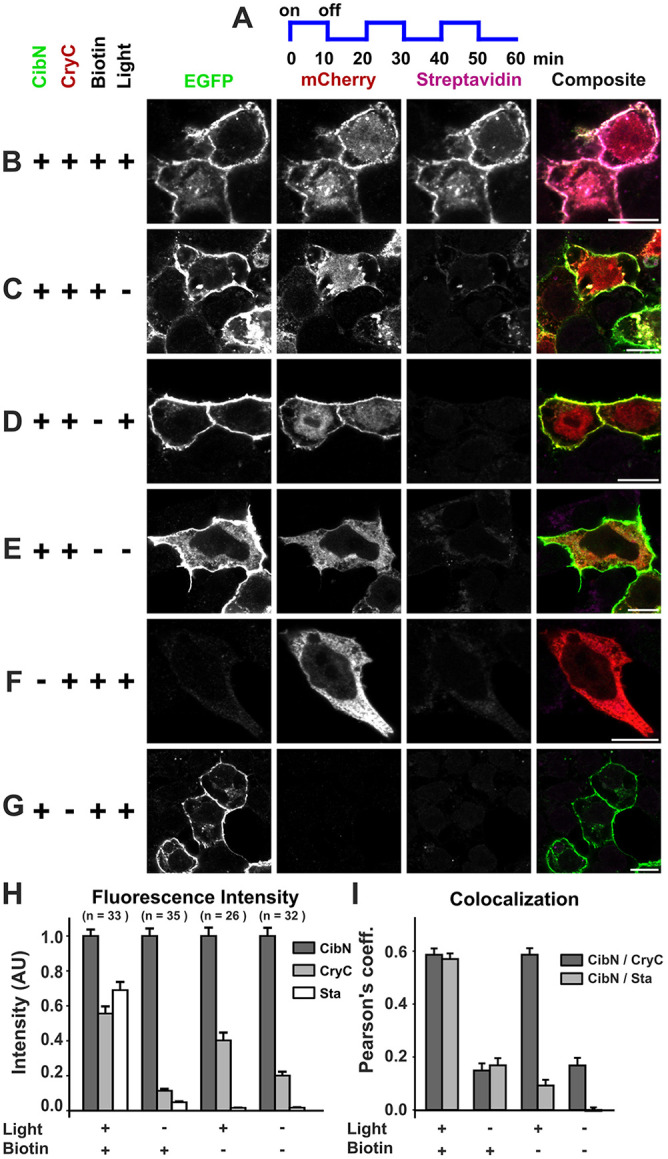
**LAB biotinylation is light-dependent.** CibN and CryC were transiently co-expressed in HEK 293T cells and, in the presence of 100 µM biotin, were exposed to 10 min cycles of alternating blue light and darkness for 1 h. The cells were fixed and stained for EGFP, mCherry, and biotin. (A) Schematic of light exposure sequence used. (B) Immunofluorescence showing that in the presence (+) of exogenous biotin and light, membrane-localized CibN (EGFP) and CryC (mCherry) dimerized and biotinylated proteins (Streptavidin, Sta) in the vicinity of the plasma membrane, with composite images confirming the colocalization of CibN, CryC, and Sta. (C–E) In the absence (−) of blue light (C), in the absence of added biotin (D) or in the absence of both biotin and light (E), no biotinylation was observed. (F,G) When only CryC (F) or CibN (G) were expressed, no biotinylation was detected, showing CibN and CryC dimerization are essential for proximity labeling. To facilitate an unbiased visual comparison of fluorescence intensities in B–G, the panels in each column display identical minimum and maximum intensities. (H) Bar graph of CryC, CibN, and Sta membrane fluorescence intensity averaged across number of cells from three biological replicates showing that there is significant biotinylation occurring only in the presence of light and biotin. CryC intensity proximal to membrane is significant only in the light-positive conditions, showing it localizes with the membrane only in the presence of light. The fluorescence intensity of CryC and Sta proximal to membrane region was normalized to CibN to account for variation in CibN expression levels in different cells. Normalized fluorescence intensities are tabulated in Table S1 and corresponding *P*-values are tabulated in Table S3. (I) Average Pearson's coefficient values showing that CibN and CryC colocalize in the presence of light, whereas Sta colocalizes to CibN only in the presence of light and biotin. Average Pearson's coefficient values are tabulated in Table S2 and corresponding *P*-values are tabulated in Table S4. Scale bars: 10 µm. Error bars are s.e.m. *n*=number of cells from three biological replicates for all conditions.

Given that split-TurboID shows significant biotin labeling after 1 h (Cho et al., 2020a), we transiently co-expressed CibN and CryC in HEK cells and exposed the cells to alternating 10-min cycles of blue light (470 nm) and darkness for 1 h using a blue LED light in the presence of 100 µM biotin (Fig. 2A). We then fixed the cells and stained them for GFP, mCherry and biotin using anti-GFP and anti-mCherry antibodies and Alexa-Fluor-647-conjugated streptavidin (Sta) (Fig. 2B–G). Next, we measured the level of CibN, CryC and Sta on the membrane using CibN as a membrane indicator for region of interest (ROI) selection (Fig. 2H; Table S1). Fluorescence intensities were normalized to CibN fluorescence intensity to account for differences in CibN expression levels in different cells. Given that CibN is membrane bound whereas CryC is expressed in the cytoplasm, there were always significantly more CryC molecules in a cell than CibN molecules. We also quantified the colocalization of CryC and Sta with membrane-bound CibN (Fig. 2I; Table S2). All *P*-values (Tables S3, S4) and raw fluorescence data (Fig. S1A) are cataloged in the supplementary information.

In the presence of light and biotin, CryC showed a high colocalization with CibN at the plasma membrane, which resulted in high Sta fluorescent intensity corresponding to significant membrane proximal biotinylation (Fig. 2B,H,I; Tables S1, S2). When samples were kept in darkness, the CryC did not localize to the membrane and no biotinylation was observed (Fig. 2C,H,I; Tables S1, S2). Similarly, when cells were exposed to light in the absence of biotin, CryC had significant membrane fluorescence intensity and colocalization with CibN (Fig. 2D,H,I; Table S2), but did not result in significant biotinylation, as Sta fluorescent intensity was very low at the membrane with negligible colocalization between Sta and CibN (Fig. 2H,I; Table S2). Furthermore, in the absence of both light and biotin, very low membrane CryC and Sta fluorescence intensity and CibN colocalization were measured, corresponding to negligible biotinylation (Fig. 2E,H,I; Tables S1, S2). Finally, no biotinylation was measured when only CryC (Fig. 2F) or CibN (Fig. 2G) was expressed. Hence, the immunofluorescence data demonstrate that CibN and CryC dimerize and biotinylate only when exposed to blue light. Using the measured Pearson's coefficients of 0.59 and 0.15 for the L+/B+ and L−/B+ (L, light; B, biotin) conditions, respectively (Table S2), we calculated the coefficients of determination (the squares of the Pearson's coefficients), as 0.35 and 0.02. These coefficients of determination demonstrate that ∼35% of CryC localization to the membrane upon light exposure can be attributed to the presence of CibN, whereas only ∼2% of CryC localization to the membrane in the absence of light is dependent on the presence of CibN. This indicates a greater than 15-fold increase in interactions between CibN and CryC due to light exposure.

### Ecad–LAB activity is light dependent

**Fig. 3.**
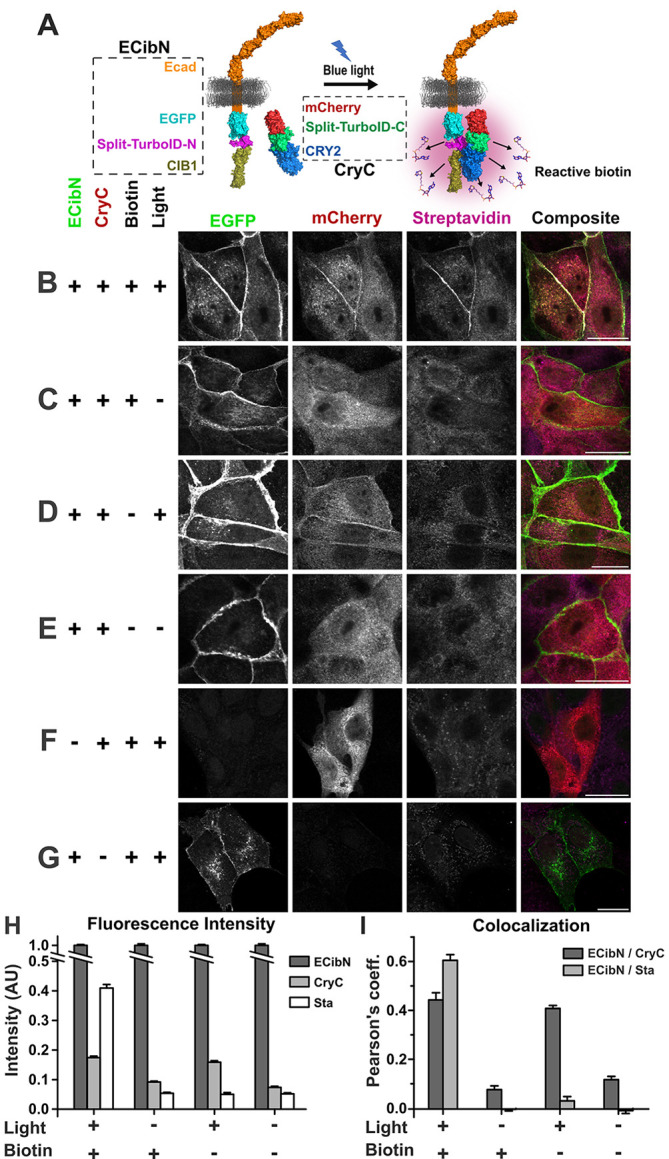
**Ecad–CibN and CryC dimerize and biotinylate only when exposed to light.** (A) Schematic of Ecad–LAB. Split-TurboID (-N), and CIB1 were fused onto the C-terminus of Ecad–EGFP to create ECibN. CryC was expressed in the cytoplasm. Both constructs were stably co-expressed in MDCK cells. When exposed to blue light, dimerization of ECibN and CryC reconstitutes the split-TurboID, which generates reactive biotin for proximity labeling. (B) Cells incubated with 100 µM biotin were illuminated with blue light, for an hour, on a 10 min on–off cycle. The cells were fixed and stained for EGFP, mCherry and biotin. ECibN localizes to the intercellular junction, and in the presence (+) of light and biotin dimerizes with CryC resulting in the biotinylation of proteins close to the membrane, which is confirmed with composite images showing the colocalization of CibN, CryC, and Sta. (C–E) In the absence (−) of light (C), biotin (D) or both (E), no biotinylation is observed. (F,G) When only CryC (F) or ECibN (G) were transiently expressed in MDCK cells, no biotinylation was observed. To facilitate an unbiased visual comparison of fluorescence intensities in B–G, the panels in each column display identical minimum and maximum intensity. (H) Membrane fluorescence intensity of CryC, ECibN and Sta, normalized to ECibN intensity to account for variation in expression levels in different cells, shows that CryC only associates to the membrane in light and biotinylation is detected only in the presence of light and biotin. Normalized fluorescence intensities are tabulated in Table S5 and corresponding *P*-values are tabulated in Table S7. (I) Average Pearson's coefficient values showing that ECibN and CryC colocalize in the presence of light, whereas Sta colocalizes to ECibN only in the presence of light and biotin. Average Pearson's coefficient values are tabulated in Table S6 and corresponding *P*-values are tabulated in Table S8. Scale bars: 20 µm. Error bars are s.e.m. *n*=156 cells evenly split between three biological replicates for all conditions.

Next, to validate LAB activity with a well-characterized protein and in a different cell line, we fused LAB to the ubiquitous, transmembrane protein E-cadherin (Ecad) in MDCK cells. Ecad is an essential cell–cell adhesion protein that plays key roles in the formation and maintenance of epithelial tissues and acts as a tumor suppressor (Xie et al., 2022). Besides binding homophilically, Ecad also has many recorded heterophilic transmembrane binding partners such as the desmosomal adhesion proteins desmoglein-2 (Dsg2) and desmocollin-3 (Dsc3) (Shafraz et al., 2020). We fused CIB1 and spTN onto Ecad–EGFP (ECibN) (Fig. 3A) and stably expressed it with CryC in MDCK cells containing endogenous Ecad.

First, we confirmed that Ecad–CibN localizes to cell–cell junctions (Fig. 3B). Next, cells were exposed to alternating 10 min cycles of blue light and darkness for 1 h before fixing and immunostaining, and CryC accumulation at the junction was monitored. To measure biotinylation and localization, ECibN, CryC and Sta fluorescence intensity (normalized by ECibN fluorescence intensity to account for variation in ECibN expression levels), were measured on the cell membranes; ECibN was used as a membrane indicator for ROI selection. The high CryC and Sta fluorescence intensity near the membrane in the presence of light and biotin confirmed biotinylation activity, with high colocalization coefficients for ECibN with both CryC and Sta (Fig. 3B,H,I; Tables S5, S6). In the absence of light and the presence of exogenous biotin, CryC did not colocalize with ECibN, and a low membrane intensity and colocalization coefficient was measured (Fig. 3C,H,I; Tables S5, S6). Additionally, no biotinylation was detected, with a negligible Sta membrane intensity and colocalization coefficient between ECibN and Sta (Fig. 3C,H,I; Tables S5, S6). This indicates that Ecad–LAB biotinylation is light dependent. Furthermore, when cells were illuminated with blue light in the absence of biotin, even though ECibN and CryC showed colocalization and CryC had an increased membrane intensity, the relative Sta intensity and colocalization was not significant (Fig. 3D,H,I; Tables S5, S6). Similarly, in the absence of both light and biotin, ECibN and CryC did not colocalize or show a substantial CryC or Sta membrane signal, with no colocalization between ECibN and Sta (Fig. 3E,H,I; Tables S5, S6). Finally, when only CryC (Fig. 3F) or ECibN (Fig. 3G) was expressed, there was no biotinylation observed. Thus, the immunofluorescence data for ECibN and CryC in MDCK cells confirm that LAB can be applied to other proteins of interest, in different cell lines, and that when exogenous biotin is provided, its biotinylation activity depends only on exposure to light. All *P*-values (Tables S7, S8) and non-normalized membrane fluorescence data (Fig. S1B) are cataloged in the supplementary information. It is important to note that the presence of a high background in the Sta channel for MDCK cells was due to a low ECibN expression level and not due to off-target biotinylation given that the background levels for the biotin positive and negative conditions were similar (Fig. 3B–E).

Using the measured Pearson's coefficients of 0.44 and 0.08 for the L+/B+ and L−/B+ conditions, respectively (Table S6), we calculated the coefficients of determination as 0.19 and 0.006. This demonstrates an over 20-fold increase in the interaction between ECibN and CryC between the light and dark conditions. Finally, to quantify how quickly Ecad–LAB biotinylates proximal proteins, we performed immunofluorescence analysis for MDCK cells exposed to light for different durations (1 min, 10 min and 30 min; Fig. S2). The data shows statistically significant biotinylation after just 1 min of light exposure, indicating that Ecad–LAB is indeed biotinylating on this timescale (Fig. S2A,D,E).

### Benchmarking biotinylation efficiency of LAB against stand-alone TurboID

We used western blots to benchmark the biotinylation efficiency of Ecad–LAB exposed to light for different durations (1 h, 3 h, 5 h and 18 h) against Ecad–Turbo incubated in biotin for 10 mins (Fig. S3). To prevent phototoxicity in Ecad–LAB cells due to their long exposure to blue light, a shorter on–off light cycle (1 min on: 5 min off) was used. Western blots were performed on whole-cell lysates with an equal amount of protein loaded in each lane; biotinylated proteins were stained with anti-biotin antibody conjugated to HRP.

**Fig. 4.**
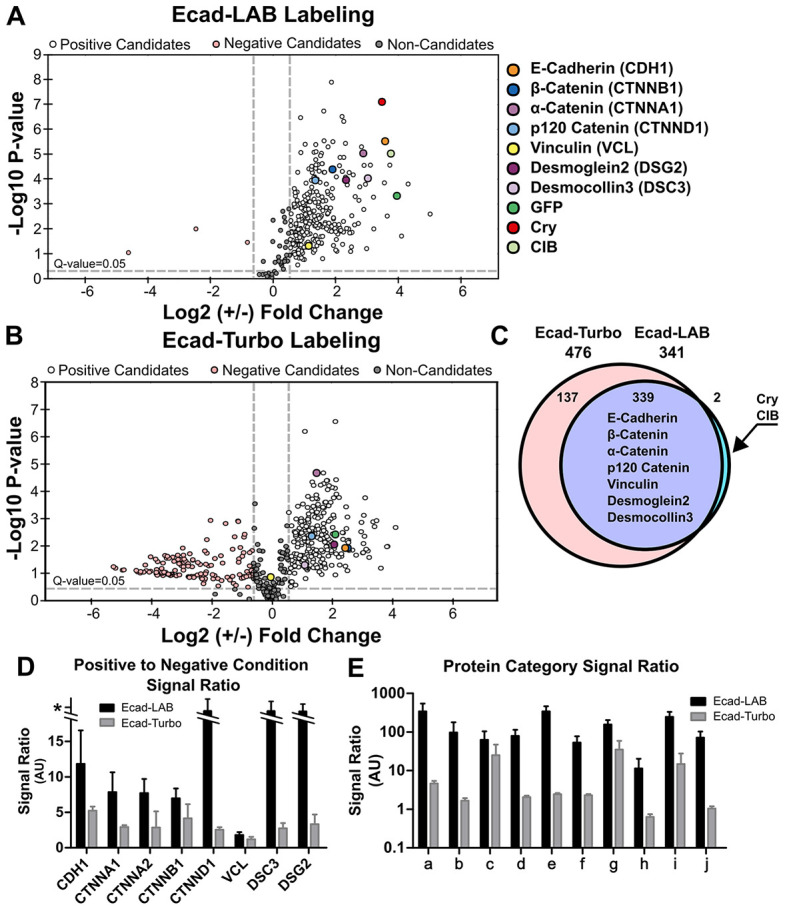
**Mass spectrometry shows that Ecad–LAB is more accurate and precise than Ecad–Turbo.** (A) Volcano plot showing the enrichment of biotinylated proteins in Ecad–LAB, in the presence (positive; white) and absence (negative; pink) of light. Known Ecad-associated proteins are labeled in different colors and are highly enriched. (B) Volcano plot showing the enrichment of biotinylated proteins in Ecad–Turbo, in the presence (positive; white) and absence (negative; pink) of biotin. There are many more negative ‘hits’ for Ecad–Turbo compared to Ecad–LAB. (C) Every hit (positive candidates, negative candidates, and non-candidates) in Ecad–LAB was also a hit in Ecad–Turbo, with the expected exceptions of Cry and CIB. (D) Comparison of positive and negative signals for several key Ecad-binding proteins highlighted in A. Ecad–LAB has a universally higher signal ratio. Proteins without any signal in the negative condition were given an arbitrarily high ratio value (*) to avoid dividing by zero. (E) Signal ratio for broad categories of proteins, graphed on a log scale. Categories were chosen based on previously published categories in the Ecad interactome (Shafraz et al., 2020), as follows: a, adhesion receptors; b, cytoskeletal components and motors; c, kinases and phosphatases; d, membrane-binding adaptors, receptors and transporters; e, other/unknown/secreted; f, actin- and microtubule-binding and dynamics-associated; g, adaptors; h, metabolic enzymes; I, chaperone, trafficking degradation and GTPase regulation; j, DNA and translation. Data in A–E are representative of three repeats for the Ecad–LAB conditions and two repeats for the Ecad–Turbo conditions. Data in D and E are mean±s.e.m.

Although Ecad–Turbo incubated with biotin for 10 mins had higher biotinylation levels than Ecad–LAB at all measured timepoints (Fig. S3A), the larger number of false positives in Ecad–Turbo (Fig. 4A,B; Fig. S4) made a direct comparison of biotinylation from western blot bands difficult. We therefore compared the biotinylation efficiency of Ecad–LAB for one time point (1 h) against Ecad–Turbo and used a single band at ∼130 kDa, which is present in the Ecad–LAB and Ecad–Turbo lanes (but not in the un-transfected control), for subsequent analysis (Fig. S3A). It is important to note that although every Ecad–Turbo protein is capable of biotinylating a target, Ecad–LAB has a Pearson's correlation coefficient of 0.44 (see Fig. 3), implying that a much smaller fraction of ECibN is bound to CryC and capable of biotinylating. Furthermore, differences in the expression levels of ECibN and Ecad–Turbo could also impact the measured levels of protein. However, even with these limitations the ratio between the positive and negative condition bands for each construct showed that Ecad–LAB with 1 h light exposure had a similar biotinylation efficiency to Ecad–Turbo incubated with biotin for 10 min (Fig. S3C). In contrast, stand-alone Split-TurboID needed 4 h to approach 1 min of full-length TurboID efficiency (Cho et al., 2020b).

### Benchmarking proteome of LAB against stand-alone TurboID

To benchmark LAB performance, we compared Ecad proteomes generated using both LAB and stand-alone full-length TurboID (Table S10). A biotinylated LAB proteome was generated using MDCK cells stably expressing Ecad–LAB. The cells were incubated in 100 µM biotin and were either exposed to blue light for 1 h using 10 min on–off cycles (positive condition) or were kept in darkness (negative condition). To generate a corresponding biotinylated TurboID proteome, MDCK cells were stably transfected with Ecad conjugated to full-length TurboID (Ecad–Turbo). The Ecad–Turbo cells were either incubated with (positive condition) or without (negative condition) 100 µM biotin for 1 h. The biotinylated proteins generated by Ecad–LAB and Ecad–Turbo were then captured from the corresponding cell lysate using streptavidin-coated magnetic beads. The captured proteins were trypsin digested before liquid chromatography tandem MS (LC-MS/MS) was performed for three replicates per condition (Ecad–LAB) or two replicates per condition (Ecad–Turbo).

The Ecad–LAB MS data showed significant enrichment of proteins in cells illuminated with blue light compared to those kept in darkness (Fig. 4A). We then compared Ecad–LAB-positive and -negative hits in the presence and absence of light against Ecad–Turbo in the presence and absence of biotin (Fig. 4). Here, ‘negative hits’ are proteins that have higher levels in the negative conditions, throwing doubt on the legitimacy of their presence in the positive conditions. This data demonstrates that Ecad–LAB had 297 positive hits and only three negative hits (Fig. 4A). In contrast, Ecad–Turbo had 241 positive hits and 137 negative hits (Fig. 4B). Well-established members of the core Ecad–catenin complex (Guo et al., 2014) such as Ecad, β-catenin (CTNNB1), α-catenin (CTNNA1), p120-catenin (CTNND1) and vinculin (VCL) were highly enriched in the Ecad–LAB data. Similarly, well-established Ecad transmembrane binding partners such as Dsg2 and Dsc3 were prominent hits in Ecad–LAB data (Shafraz et al., 2020) (Fig. 4A). Importantly, all the hits (positive candidates, negative candidates and non-candidates) in Ecad–LAB were also hits in Ecad–Turbo, with the expected exceptions of the light-activated dimer components (Cry and CIB) (Fig. 4C). This demonstrates that Ecad–LAB is at least as accurate as Ecad–Turbo, with an increased precision resulting in fewer extraneous hits.

Comparison of the ratio of positive to negative signal levels for Ecad and its key binding partners (highlighted in Fig. 4A) demonstrated that Ecad–LAB has a universally higher signal-to-background ratio compared to Ecad–Turbo (Fig. 4D). This trend extended for the entire Ecad–LAB interactome as seen in the signal-to-background ratios for broad categories of proteins (Fig. 4E). Fig. 4E shows the Ecad–LAB interactome sorted into previously published categories (Shafraz et al., 2020) as described in the figure legend. We further directly compared the biotin-negative and light-negative conditions of Ecad–Turbo and Ecad–LAB, respectively, which showed an almost universally higher absolute protein level present in the negative Ecad–Turbo condition compared to the negative Ecad–LAB condition (Fig. S4). Taken together, this data quantitatively demonstrates that LAB has a significantly lower background biotinylation than conventional TurboID.

## DISCUSSION

Given that LAB was designed to map membrane PPIs, we benchmarked its performance on the cell membrane. Using immunofluorescence, western blots and MS in both HEK293T and MDCK cells, we demonstrated that LAB can be selectively activated through researcher-controlled biotin and light application. We were able to demonstrate statistically significant biotinylation after only 1 min of light exposure, showing that functional complementation of LAB is achieved on a rapid timescale (Fig. S2A). The specificity of LAB was validated through MS analysis showing light exposure-dependent enrichment of many proteins known to bind to Ecad, including the α-catenin–β-catenin–vinculin complex that links Ecad to the actin cytoskeleton (Fig. 4A). Importantly, these and other known Ecad-binding partners were highly enriched in the light-positive condition compared to the light-negative condition, with a signal-to-background ratio an order of magnitude higher than that of Ecad-conjugated full-length TurboID under biotin-positive and -negative conditions (Fig. 4E). Additionally, not every major Ecad-binding partner was successfully detected as a positive candidate by Ecad–Turbo, possibly a result of its high background labeling reducing data resolution (Fig. 4B). Taken together, this shows that LAB successfully labels proximal proteins in a light-dependent manner with higher specificity than full-length TurboID.

We used split-TurboID as the labeling component of LAB due to its well characterized biotinylation kinetics. Similarly, we used the photodimer pair CRY2 and CIB1 because they are one of the most widely used photodimer systems, and have been tested in many different cell lines as well as in living organisms to investigate a wide variety of cellular functions (Taslimi et al., 2016). Importantly, the binding kinetics of CRY2 and CIB1 complement those of Split-TurboID by remaining associated long enough after light exposure to allow for sufficient biotinylation, while not remaining bound too long post-exposure so that the labeling reaction can be arrested with the removal of light.

It has previously been shown that CRY2 can homodimerize when illuminated with blue light (Wang and Lin, 2020). However, this is not an issue for LAB given that our construct only has the inactive C-terminus of Split-TurboID associated with CRY2. Consequently, CRY2 homodimerization cannot reconstitute a functional TurboID. This is supported by our data showing that no biotinylation is observed in the CryC-only condition (Fig. 2F). Furthermore, previous studies have shown that CRY2 homodimerization does not prevent interaction with CIB1 (Bugaj et al., 2013).

Although we have only validated LAB with cell membrane proteins, we anticipate that LAB will efficiently biotinylate target proteins in different cellular compartments. It has already been demonstrated that both split-TurboID (Cho et al., 2020b) and stand-alone TurboID (Branon et al., 2018) are capable of biotinylating target proteins in different organelles with distinct pH, redox environments and endogenous nucleophile concentrations. Additionally, because LAB relies on light rather than a diffusion-limited chemical cofactor, it can easily be activated in different cellular compartments. The main consideration will be expressing LAB sufficiently in different compartments, which is more a function of construct design than of its biotinylation ability. Importantly, truncated versions of the CRY2 and CIB1 proteins have been developed (Taslimi et al., 2016), which can be used in LAB if large constructs would affect expression and localization of bait proteins tagged with LAB.

A consideration when using LAB is the possibility of phototoxicity after long-duration exposure to the activating blue light source. Owing to binding half-life of CRY2–CIB1 being ∼5 min, alternating 10-min cycles of light and darkness allowed significant dimerization and biotinylation, while reducing phototoxicity for our shorter-term (<1 h) experiments. For longer duration experiments (see Fig. S3), we used a shorter light cycle (1 min on, 5 min off) to further reduce phototoxic effects while still maintaining maximal Ecad–LAB activation. However, even with this modified light exposure protocol, differences in intensities between biotinylated protein bands in the L+ and L− conditions decreases as the light exposure is increased beyond 1 h (Fig. S3). Consequently, after 18 h light exposure, the intensities of the L+ and L− bands are similar. It is likely that this occurs because long periods of light exposure damage the cells and brings the harvested biotinylated protein closer to the background levels. Therefore, for longer-term experiments, the optimal light intensity and exposure cycle will likely need to be determined and further optimized based on the cells and application.

Recently, a different opto-dimerization system, iLID, has been used with a new split-TurboID variant (Chen et al., 2022). However, iLID has a half-life of under a minute (Kennedy et al., 2010; Taslimi et al., 2016), which might not provide enough time for appreciable labeling to occur before the dimers unbind and inactivate Split-TurboID. Furthermore, in this opto-dimerization study, TurboID was split at a previously uncharacterized location (G99/E100) and consequently, the rate of biotinylation of this new split-TurboID variant is unknown (Chen et al., 2022).

Another PL tool using light-activated miniSOG has been recently developed (PDPL), which uses singlet oxygen to create electrophilic residues on prey proteins, which can be subsequently bound to an alkyne chemical probe and pulled down using click chemistry (Zhai et al., 2022). However, the aniline probe used with PDPL can be toxic to cells (Zhai et al., 2022; Wang et al., 2016). Furthermore, miniSOG has a labeling radius of up to 70 nm, which is much larger than the 10 nm labeling radius for TurboID (Kim et al., 2014). Although a larger labeling radius is appropriate for identifying compartmentalization of proteins, the smaller labeling radius of LAB is more suited for discovering direct PPIs (Cho et al., 2020b; Zhai et al., 2022). Another light-activated PL tool, MicroMap, which uses an antibody conjugated to an iridium photocatalyst to label nearby proteins via carbene intermediates, has also recently been developed (Geri et al., 2020; Seath et al., 2021). However, the necessity of a primary antibody that binds to the bait protein at a convenient location limits applications for MicroMap.

Finally, another light-based biotinylation tool called LOV-Turbo, which is a fusion between the light-activated LOV domain and TurboID, was recently developed (Lee et al., 2023). Although LOV-Turbo is simpler to use than LAB since it requires transfection with only one construct, its ability to accurately and precisely determine the interactome of a bait protein has not been comprehensively demonstrated (Lee et al., 2023). Consequently, LOV-Turbo might be preferable for cellular compartment-based searches, such as identifying proteins that traffic between specific cell compartments, whereas LAB is intended for proteome searches centered around a single bait protein of interest.

Unlike CRY2 and CIB1, which photodimerize in the presence of blue light, photodimerization activated by longer wavelength light could be more effective for PPI identification in deeper tissue. However, the two known red light activated photo-hetrodimers (PhyB and PIF3, and PhyB and PIF6), require light exposure at different wavelengths for both activation and inactivation (Klewer and Wu, 2019). We therefore chose a photodimer pair that dissociated in the dark, rather than through alternate wavelength exposure, for ease of use. Importantly, since CRY2–CIB1 dimerization can be triggered using two-photon excitation (Kennedy et al., 2010), this might allow LAB to map PPIs in deeper tissue *in vivo*.

We anticipate that the ability to activate LAB using light, coupled with its high temporal resolution, can be exploited to interrogate differences in membrane PPIs at different time points in the cell cycle. We also expect that by using focused activating light, LAB can be used to identify differences in PPIs at distinct cellular locations. Finally, like TurboID, which has previously been used to map extracellular PPIs (Shafraz et al., 2020), we anticipate that LAB can also be used to investigate extracellular protein interactions with high spatial and temporal resolution.

## MATERIALS AND METHODS

### Cloning of plasmid constructs

CibN was generated by restriction digesting a CIB-pmEGFP plasmid (Addgene plasmid #28240; Kennedy et al., 2010) at AgeI and NheI sites and fusing PCR amplified CIB1 and spTN (Addgene plasmid #153002; Cho et al., 2020a) using Gibson assembly. SpTN was connected to CIB1 and pmEGFP with linkers V5-KGSGSTSGSGTG (Linker 1) and GSGPVAT. CryC was constructed by inserting PCR amplified spTC (Addgene plasmid #153003; Cho et al., 2020a) onto restriction-digested Cry2-mCherry (Addgene plasmid #26871; Kennedy et al., 2010) at XmaI sites with linkers ARGKGSGSTSGSG and KGSGDPPVAT.

To design ECibN, V5-spTN-CIB-pmEGFP was constructed by restriction digesting CIB1-pmEGFP with NheI and inserting PCR amplified Linker1-spTN-KGSGAT. Then, the Ecad–EGFP plasmid was restriction digested at NotI and XhoI sites and EGFP was reintroduced without the stop codon. Next, the NotI and HindIII sites of Ecad–EGFP were restriction digested and PCR-amplified V5-spTN-CIB was inserted using Gibson assembly. Ecad–EGFP plasmid was a kind gift from Prof. Soichiro Yamada at the University of California, Davis, CA, USA. All enzymes were high efficiency enzymes from New England Biolabs. All primers used are listed in Table S9.

For development of a dual-expressing stable Ecad–LAB cell line, a blasticidin (BSD)-resistant CryC plasmid was generated by replacing spTN from Addgene plasmid #153002 (Cho et al., 2020a) using BstBI and NheI enzyme digestion and inserting CryC using Gibson assembly.

### Cell culture and transfection

HEK293T cells (ATCC, CRL-11268) were grown in high-glucose (4.5 g/l) Dulbecco's modified Eagle's medium (DMEM, Gibco) cell culture medium with 10% fetal bovine serum (Gibco) and 1% penicillin-streptomycin (PSK) (10,000 U/ml, Life Technologies). MDCK cells (ATCC) were cultured in low glucose (1 g/l) DMEM (Gibco) cell culture media with 10% fetal bovine serum and 1% PSK.

HEK293T cells were transiently transfected at 80% confluency with CibN and CryC plasmids using polyethylenimine (PEI) (Polysciences, Inc.). Plasmids and PEI were diluted in Opti-MEM (Gibco) at a 1µg:5 µg DNA:PEI ratio. After 5 min, both solutions were mixed and incubated for 90 min and then added to the cells. In order to develop the MDCK stable lines, cells were transfected with the ECibN and BSD-CryC plasmids at 30% confluency using lipofectamine 3000 (Invitrogen) with the same DNA ratio. After 24 h, the cells were passaged and sparsely seeded onto p150 dishes. After allowing a further 24 h for cell attachment and construct expression, 500 µg/ml G418 (Gibco) and 5 µg/ml BSD (Corning) were added to the medium for antibiotic selection. Colonies were picked after they reached ∼2 mm in size and transferred to a 96-well plate. Colonies were fluorescently imaged to detect expression, and the top co-expressing colonies were further expanded. Expanded colonies were further imaged, and the brightest clone with the most uniform expression was chosen for experiments.

### Live-cell imaging

HEK cells were imaged >12 h post-transfection using a 561 nm laser to visualize mCherry and a 488 nm laser to image EGFP and to photo-excite CryC. Images were reconstructed using ImageJ. For the intensity profile of CryC accumulation, a 1 µm line was drawn across a selected membrane region and the raw pixel intensity across the line was plotted (Fig. 1C). Measurements were taken using Plot Profile analysis feature in ImageJ. To plot CryC accumulation over time, a 0.225 µm by 0.900 µm box was drawn on the cell membrane bracketing the intensity profile line (Fig. 1C). Measurements for each frame were taken using the ROI mean intensity measurement capability in ImageJ.

### Immunofluorescence

Cells were transferred to low glucose, phenol red-free DMEM (Gibco) with 10% FBS, incubated with 100 µM biotin and exposed to blue light in 10 min intervals for 1 h using a blue LED light source (Blue box Pro Transilluminator, miniPCR bio). Control cells were kept in dark. Then, cells were immediately fixed using 3% paraformaldehyde and 0.3% Triton X-100 in phosphate-buffered saline (PBS) for 10 min and blocked with 1% bovine serum albumin (BSA) and 0.3% Triton X-100 in PBS for 30 min. Anti-GFP antibody (rabbit polyclonal, Rockland Immunochemicals) (HEK293T cells), anti-GFP antibody (chicken polyclonal, Rockland Immunochemicals) (MDCK cells), Alexa Fluor 488-conjugated goat anti-rabbit-IgG antibody (Life Technologies) (HEK293T cells), and Alexa Fluor 488-conjugated goat anti-chicken-IgY antibody (Invitrogen) (MDCK cells) were used to detect GFP-tagged proteins. Anti-mCherry (mouse monoclonal, Invitrogen) (HEK293T cells), anti-mCherry (rabbit monoclonal, Invitrogen) (MDCK cells), Alexa Fluor 568-conjugated goat anti-mouse-IgG antibody (Invitrogen) (HEK293T cells) and Alexa Fluor 568-conjugated goat anti-rabbit-IgG (Invitrogen) (MDCK cells) were used to detect mCherry. Biotinylated proteins were identified with Alexa Fluor 647-conjugated streptavidin (Invitrogen). Primary antibodies were incubated for 1 h (1:1000 dilution in PBS with 1% BSA and 0.3% Triton X-100) and secondary antibodies were incubated for 30 min (1:1000 dilution in PBS with 1% BSA and 0.3% Triton X-100). Cells were imaged using a Leica Microsystems Stellaris 5 confocal setup with 63×/1.40 NA oil objective. Images were reconstructed using ImageJ.

### Image analysis

All analysis was undertaken in ImageJ. In order to reduce background and oversaturated pixel contribution, all images were processed with a Gaussian filter with a sigma of 11 (HEK images) or 15 (MDCK images). The filtered image was then subtracted from the original image. CibN or ECibN expression was used to determine the area to be measured, and a threshold mask was created using the CIB–GFP signal using a threshold minimum of 50 on the Threshold tool. Membrane ROIs were hand-chosen with the wand tool from the CibN or ECibN GFP channel using this mask. As CibN and ECibN localize to the cell membrane, the ROIs only contain the plasma membrane. Higher Gaussian sigmas were used on MDCK cells due to the lower expression of ECibN. The ROIs were used for both fluorescence intensity measurements and colocalization analysis. Colocalization analysis was carried out on the processed images using the Coloc2 plugin with Costes threshold regression, a point spread function (PSF) of 3, and 10 Costes randomizations. Unthresholded Pearson's coefficients were used from the outputs. Intensity measurements for all channels were taken on the processed images using the Multi Measure functionality on the ROI manager.

### Sample preparation for MS and western blot analysis

MDCK cells stably expressing Ecad–LAB or Ecad–Turbo and HEK cells transiently transfected with LAB were cultured on p150 dishes (VWR). The cells were incubated with 100 μM biotin and exposed to blue light on a 10 min on/off cycle for 1 h (LAB, Ecad–LAB) or exposed to 100 μM biotin alone (Ecad–Turbo). Control cells were kept in darkness (LAB, Ecad–LAB) or without exogenous biotin (Ecad–Turbo); ∼8×10^7^ cells were used for each replicate. After incubation, cells were washed three times with PBS, scraped and centrifuged (775 ***g*** for 15 min). Pelleted MDCK cells were resuspended in M2 lysis buffer (50 mM Tris-HCl pH 7.5, 150 mM NaCl, 1% SDS and 1% Triton X-100; Muinao et al., 2018) with 5 μl/ml of protease inhibitor mixture (Sigma-Aldrich) and 1 μl/ml benzonase nuclease (250 U/μl; Millipore-Sigma). Pelleted HEK293T cells were resuspended in lysis buffer [50 mM Tris-HCl pH 7.5, 150 mM NaCl, 0.4% SDS, 1% octyl phenoxy poly(ethyleneoxy) ethanol (IGEPAL CA-630), 1.5 mM MgCl_2_ and 1 mM EGTA] with 2 μl/ml of protease inhibitor mixture (Sigma-Aldrich), and 1 μl/ml benzonase nuclease (250 U/μl) (Millipore-Sigma). The lysate was flash frozen in dry ice and quickly thawed at 37°C. The lysate was then incubated for 30 min at 4°C and sonicated at 30% duty ratio for 1 min. Next, the lysate was centrifuged at 14,549 ***g*** for 30 min at 4°C. After that, the concentrations of the supernatant were measured with an RC DC protein assay kit (Bio-Rad). Supernatant concentration was adjusted to the lowest concentration and lysis buffer was added to bring all volumes to 1 ml. Supernatant was incubated with 50 μl (HEK293T) or 100 μl (MDCK) of superparamagnetic streptavidin-conjugated beads (Dynabeads MyOne Streptavidin C1, Invitrogen) and rotated overnight at 4°C. The next day, beads were washed with the lysis buffer. Then, beads were washed with the 2% SDS in 50 mM Tris-HCl pH 7.4, and twice with lysis buffer.

For MS sample preparation, magnetic beads loaded with biotinylated proteins were washed three times with 50 mM ammonium bicarbonate (NH_4_HCO_3_) and resuspended in 60 μl of 50 mM NH_4_HCO_3_ containing 5 µg of trypsin for overnight digestion at 37°C in a shaker. The resulting peptides were recovered from the beads by removing the supernatant after pelleting the beads via magnetic holder (Cell Signaling Technology), and tryptic peptides from the supernatant were dried in a vacuum centrifuge and reconstituted in 0.1% formic acid. Tryptic peptides were analyzed using nano-scale liquid chromatographic tandem mass spectrometry (nLC-MS/MS).

For MDCK western blots, whole-cell lysate was used, with an identical amount of total protein loaded in each lane, determined using DC assay concentration measurements. Lysate was run on a 4–15% Mini-Protean TGX Precast Protein Gel (Bio-Rad) before being transferred to a nitrocellulose membrane and stained for biotin using HRP-conjugated anti-biotin antibody (1:1000 dilution, cat. no 7075, Cell Signaling Technology). WesternBright ECL HRP substrate (Advansta) was used to detect the protein. Images were acquired using Image Lab software from Bio-Rad.

### Mass spectrometry

For each sample, an equal volume of peptide was loaded onto a disposable Evotip C18 trap column (Evosep Biosystems, Denmark) as per the manufacturer's instructions. Briefly, Evotips were wetted with 2-propanol, equilibrated with 0.1% formic acid, and then loaded using centrifugal force at 1200 ***g***. Evotips were subsequently washed with 0.1% formic acid, and then 200 μl of 0.1% formic acid was added to each tip to prevent drying. The tipped samples were subjected to nanoLC on an Evosep One instrument (Evosep Biosystems). Tips were eluted directly onto a PepSep analytical column, dimensions: 8 cm×150 µm, C18 column with 1.5 μm particle size (PepSep, Denmark), and a ZDV spray emitter (Bruker Daltronics). Mobile phases A and B were water with 0.1% formic acid (v/v) and 80/20/0.1% ACN/water/formic acid (v/v/v), respectively. The standard pre-set method of 60 samples-per-day was used, which is a 26 min gradient.

Mass spectrometry was performed on a hybrid trapped ion mobility spectrometry-quadrupole time of flight mass spectrometer (timsTOF Pro, Bruker Daltonics) with a modified nano-electrospray ion source (CaptiveSpray, Bruker Daltonics), as described previously (Shafraz et al., 2020). Mass spectrometry raw files were processed with MsFragger (Yu et al., 2020) as previously described (Shafraz et al., 2020). Volcano plots were generated using Spectronaut viewer. Known contamination proteins (Hodge et al., 2013) were removed, with further contamination filtering done by removing all non-canine proteins, with the exception of Cry and CIB from Arabidopsis (Ecad–LAB samples) and GFP from Aequorea (both), and filtering out all proteins categorized as ‘universal contaminants’ by the software. A global background signal was added using the background signal imputation method, where the software chooses the best background signal to report as the precursor quantity. This allowed protein concentrations to be compared between the light-positive and light-negative conditions and ameliorated issues where proteins at too low concentrations in the negative condition could not be compared to the light positive condition. Only proteins that had two or more unique peptides detected were included in analysis. A Q-value of 0.05 was used to determine candidate threshold. Ecad–LAB runs and Ecad–Turbo runs were analyzed in separate files.

## Supplementary Material

10.1242/joces.261430_sup1 Supplementary information

Table S10. Hits (positive candidates, negative candidates, and non-candidates) for Ecad-LAB and Ecad-Turbo
