# Supplementary material for: Light-activated BioID – an optically activated proximity labeling system to study protein–protein interactions
Source: J Cell Sci. 2023 Oct 11;136(19):jcs261430. doi: 10.1242/jcs.261430 (PMC10656424; doi:10.1242/jcs.261430)
Supplement: Supplementary information [file joces-136-261430-s1.pdf]

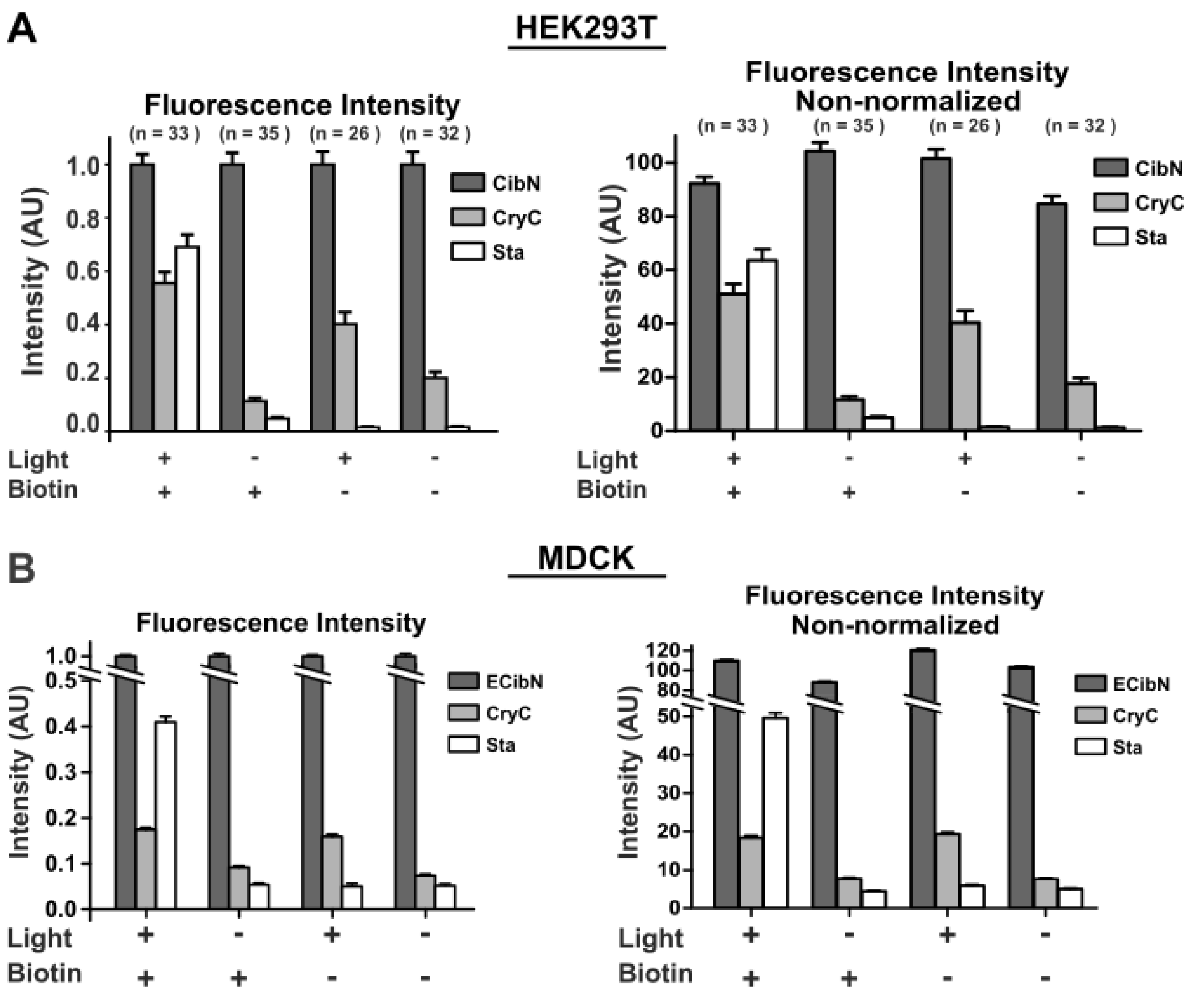

Fig. S1. Comparison of normalized and raw fluorescence intensity data. (A) HEK293T cell membrane fluorescence intensity for CibN, CryC, and Sta averaged across three biological replicates normalized to CibN membrane fluorescence (Left) or presented as measured (Right). n listed in graph, split between three biological replicates. (B) MDCK cell membrane fluorescence intensity for CryC, ECibN, and Sta, normalized to ECibN intensity (Left) or presented as measured (Right). n=156 for all conditions, evenly split between three biological replicates.

Journal of Cell Science • Supplementary information

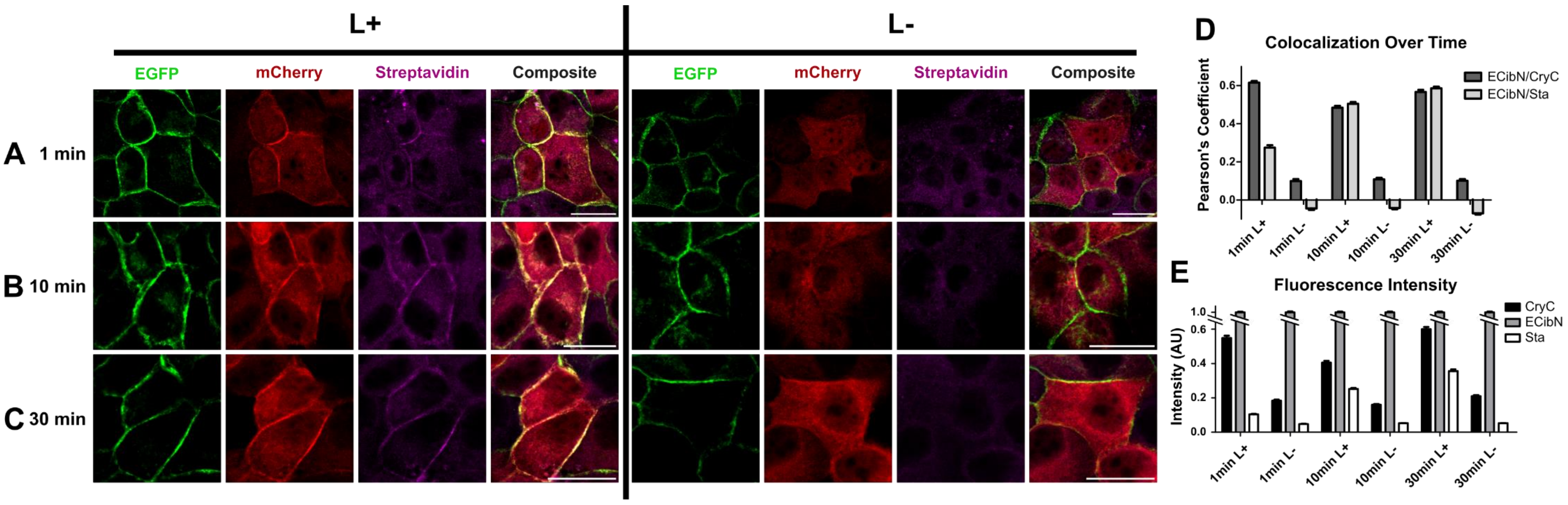

Fig. S2. Biotinylation time dependence.
Immunofluorescence images of Ecad-LAB cells incubated with 100 µM biotin, after exposure to light for (A) 1 min, (B) 10 min, and (C) 30 min. Cells were stained for GFP (green), mCherry (red), and streptavidin (magenta). Light positive (L+) images are on the left, while light negative (L-) images are on the right. The panels in each column display identical minimum and maximum intensities. Scale bars are 20 µm. For all measured light exposures, membrane localization of CryC and biotinylation was detected compared to the L- condition. D) Average Pearson's coefficients shows that ECibN and CryC colocalize in the presence of light at all time points, while Sta colocalizes to ECibN with increasing efficiency over time. E) Membrane fluorescence intensity of CryC, ECibN, and Sta, normalized to ECibN intensity to account for variation in expression levels in different cells, shows that CryC only associates to the membrane in light. Biotinylation is detected above background after only one minute and continues to increase in intensity over time. P-values for Sta signal between L+ and L- conditions are as follows: 1 min: 4.07E-29; 10 min: 1.69E-91; 30 min: 6.13E-78. Errors: s.e., n's are as follows: 1 min L+: 169; 1 min L-: 137; 10 min L+: 191; 10 min L-: 222; 30 min L+: 204; 30 min L-: 192, split between three biological replicates for all conditions.

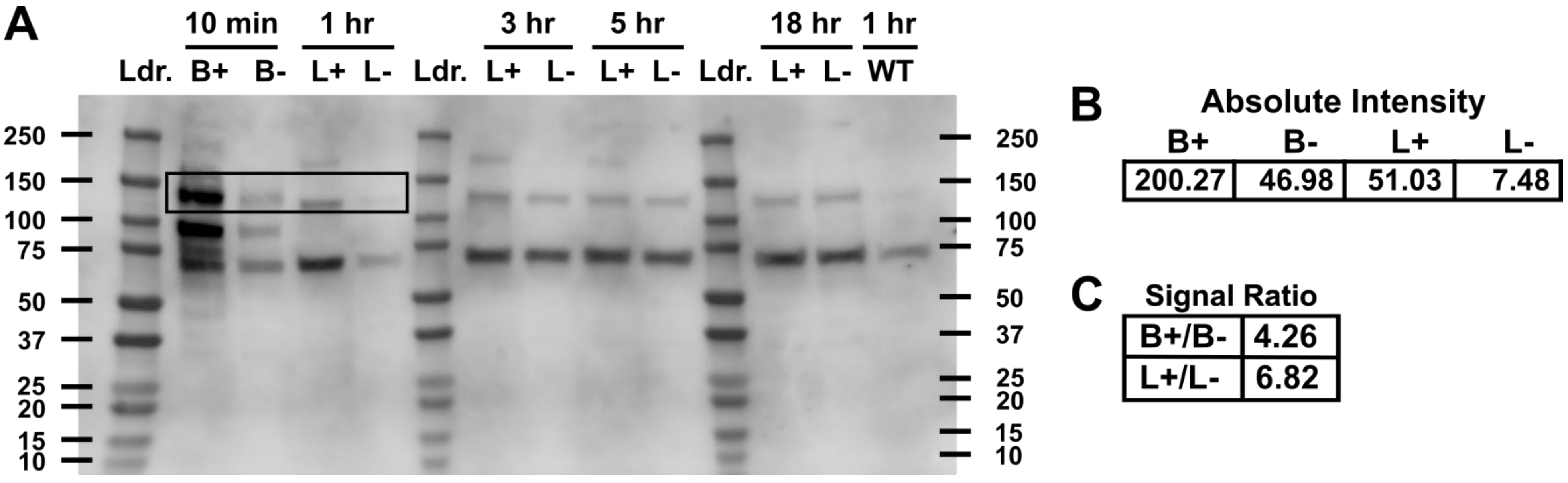

Fig. S3. Western Blots comparing Ecad-LAB and Ecad-Turbo biotinylation efficiency. (A) Western Blots comparing biotinylation efficiencies of Ecad-Turbo and Ecad-LAB expressed in stabilized WT MDCK cells for different time points. Ecad-Turbo was exposed to either the presence (B+) or absence (B-) of 100µM biotin for 10 minutes. Ecad-LAB was incubated in 100µM biotin, in the presence (L+) or absence (L-) of light for 1 hr, 3 hrs, 5 hrs, and 18 hrs, respectively. Whole lysate was stained with anti-biotin antibody. Final lane (WT) is lysate from un-transfected cells. The same amount of total protein was loaded into each lane. While Ecad-Turbo has a markedly higher biotinylation efficiency than Ecad-LAB, the larger number of false positives in Ecad-Turbo (see Fig. 4 and S3) coupled with the less than 100% co-occurrence of CryC and ECibN (Pearson's correlation coefficient of 0.44; see Fig. 3) makes a direct comparison of biotinylation efficiencies difficult. We therefore compared the biotinylation efficiency of a representative band from the first four lanes (marked by the square box). (B) Intensities of the indicated bands measured using imageJ. (C) The ratio between the positive and negative condition bands for each construct shows that Ecad-LAB exposed to light for 1 hour has a higher biotinylation efficiency than Ecad-Turbo incubated with biotin for 10 minutes. L+ cells were exposed to an altered light cycle of 1min on, 5min off to reduce phototoxicity in longer time conditions.

Journal of Cell Science • Supplementary information

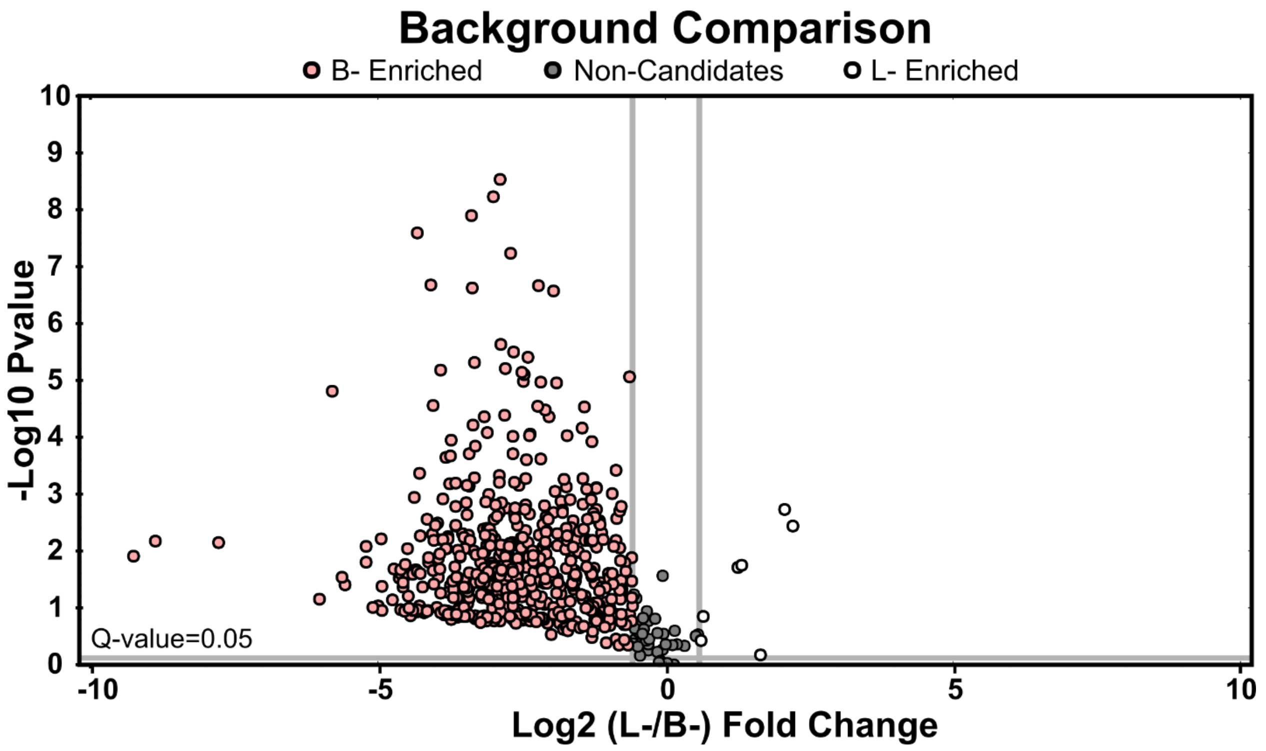

Fig. S4. Ecad-Turbo and Ecad-LAB Background. Volcano plot showing the relationship between the detected levels of protein in the Ecad-Turbo biotin negative condition (B-; pink) and the Ecad-LAB light negative condition (L-; white). Pink data points had higher protein levels in the Ecad-Turbo negative control, while white data points had higher protein levels in the Ecad-LAB negative control. Grey data points had similar levels in both conditions.

Table S1. Normalized fluorescent intensity for HEK cells

Normalized Fluorescent Intensity ± s.e. (AU)		
L+ B+	CibN	1.000 ± 0.037
	CryC	0.556 ± 0.044
	Sta	0.69 ± 0.047
L- B+	CibN	1.000 ± 0.043
	CryC	0.114 ± 0.012
	Sta	0.048 ± 0.005
L+ B-	CibN	1.000 ± 0.048
	CryC	0.402 ± 0.047
	Sta	0.016 ± 0.001
L- B-	CibN	1.000 ± 0.047
	CryC	0.201 ± 0.025
	Sta	0.017 ± 0.002

Table S2. Pearson's Coefficient for HEK cells

Pearson's Coefficient ± s.e.		
L+ B+	CibN / CryC	0.59 ± 0.02
	CibN / Sta	0.57 ± 0.02
L- B+	CibN / CryC	0.15 ± 0.03
	CibN / Sta	0.17 ± 0.03
L+ B-	CibN / CryC	0.59 ± 0.02
	CibN / Sta	0.09 ± 0.02
L- B-	CibN / CryC	0.17 ± 0.03
	CibN / CryC	-0.00 ± 0.01

Table S3. P Values for HEK cell Fluorescence

Fluorescence P Values (Two-tailed T Test)		
L+B+/ L-B+	CryC	9.08E-13
	Sta	7.78E-17
L+B+/ L+B-	CryC	0.004
	Sta	3.66E-13
L+B+/ L-B-	CryC	1.12E-08
	Sta	5.70E-17

Journal of Cell Science • Supplementary information

Table S4.P Values for HEK cell Pearson's Coefficients

Pearson's Coefficient P Values (Two-tailed T Test)		
L+B+/ L-B+	CibN / CryC	2.62E-15
	CibN / Sta	4.55E-13
L+B+/ L+B-	CibN / CryC	0.77
	CibN / Sta	1.35E-13
L+B+/ L-B-	CibN / CryC	3.35E-11
	CibN / Sta	2.83E-20

Table S5. Normalized fluorescent intensity for MDCK cells

Normalized Fluorescent Intensity ± s.e. (AU)		
L+B+	ECibN	1.000 ± 0.011
	CryC	0.174 ± 0.005
	Sta	0.410 ± 0.012
L-B+	ECibN	1.000 ± 0.015
	CryC	0.092 ± 0.003
	Sta	0.054 ± 0.002
L+B-	ECibN	1.000 ± 0.012
	CryC	0.159 ± 0.005
	Sta	0.050 ± 0.002
L-B-	ECibN	1.000 ± 0.014
	CryC	0.074 ± 0.002
	Sta	0.052 ± 0.002

Journal of Cell Science • Supplementary information

Table S6. Pearson’s Coefficient for MDCK cells

Pearson's Coefficient		
L+B+	CibN / CryC	0.44 ± 0.03
	CibN / Sta	0.61 ± 0.02
L-B+	CibN / CryC	0.08 ± 0.02
	CibN / Sta	0.00 ± 0.01
L+B-	CibN / CryC	0.41 ± 0.01
	CibN / Sta	0.03 ± 0.02
L-B-	CibN / CryC	0.12 ± 0.01
	CibN / Sta	-0.01 ± 0.01

Table S7. P Values for MDCK cell Fluorescence

Fluorescence P Values (Two-tailed T Test)		
L+B+/ L-B+	CryC	7.86E-35
	Sta	3.41E-67
L+B+/ L+B-	CryC	0.03
	Sta	2.83E-67
L+B+/ L-B-	CryC	8.33E-43
	Sta	3.60E-69

Journal of Cell Science • Supplementary information

Table S8. P Values for MDCK cell Pearson's Coefficients

Pearson's Coefficient P Values (Two-tailed T Test)		
L+B+/ L-B+	ECibN / CryC	1.22E-64
	ECibN / Sta	2.40E-112
L+B+/ L+B-	ECibN / CryC	0.01
	ECibN / Sta	3.21E-107
L+B+/ L-B-	ECibN / CryC	3.23E-56
	ECibN / Sta	4.26E-111

Table S9. List of primers used for PCR amplification

CIB1	Forward (F)	5'-ttagtgaaccgtcagatccgctagcccATGAATGGAGCTATAGGAG
	Reverse (R)	5'-tgccgatatcTACTCCTAAATTGCCATAGAG
spTurboID N	F	5'-tttaggagtagatatcGGCAAGCCCATCCCCAAC
	R	5'-cttgctcaccatggtggcgaccggtccactcccCAGAATCTGTTTAGCGTTCAGCAG
spTurboID C	F	5'-ggaaaaaatggttgcaaagcccggggtAAGGGCTCGGGCTCGACC
	R	5'-atggtggcgaccggtggatcgcccgagcccttCTTTTCGGCAGACCGCAGAC
EGFP	F	5'-tggaggtggcgaggacgacctcgagATGGTGAGCAAGGGCGAG
	R	5'-tgattatgatctagagtcgcggccgcttagaagcttgaCTTGTACAGCTCGTCCATGC
V5_spTN	F	5'-ttagtgaaccgtcagatccgctagcatgGGCAAGCCCATCCCCAAC
	R	5'-tagctccattcatggtggcgcccgagcccttCAGAATCTGTTTAGCGTTCAGCAG
spTNCIB	F	5'-tggacgagctgtacaagtcaagcttcGGCAAGCCCATCCCCAAC
	R	5'-tgattatgatctagagtcgcggccgcttaTACTCCTAAATTGCCATAGAGATTCTGC

Table S10. Hits (positive candidates, negative candidates, and non-candidates) for Ecad-LAB and Ecad-Turbo

Available for download at https://journals.biologists.com/jcs/article-lookup/doi/10.1242/jcs.261430#supplementary-data

Journal of Cell Science • Supplementary information
