# Supplementary material for: Light-activated BioID – an optically activated proximity labeling system to study protein–protein interactions
Source: J Cell Sci. 2023 Oct 11;136(19):jcs261430. doi: 10.1242/jcs.261430 (PMC10656424; doi:10.1242/jcs.261430)
Supplement: Supplementary file 1 [file joces-136-261430_review_history.pdf]

Journal of Cell Science

Light-activated BioID - an optically activated proximity labeling system to study protein-protein interactions

Omer Shafraz, Carolyn Marie Orduno Davis and Sanjeevi Sivasankar
DOI: 10.1242/jcs.261430

Editor: John Heath

Review timeline
Original submission: 21 June 2023
Editorial decision: 17 July 2023
First revision received: 10 August 2023
Accepted: 8 September 2023

Original submission

First decision letter

MS ID#: JOCES/2023/261430

MS TITLE: Light Activated BioID (LAB): an optically activated proximity labeling system to study protein-protein interactions.

AUTHORS: Omer Shafraz, Carolyn Marie Orduno Davis, and Sanjeevi Sivasankar
ARTICLE TYPE: Tools and Resources

We have now reached a decision on the above manuscript.

To see the reviewers' reports and a copy of this decision letter, please go to: https://submit-jcs.biologists.org and click on the 'Manuscripts with Decisions' queue in the Author Area. (Corresponding author only has access to reviews.)

As you will see, the reviewers raise a number of criticisms that prevent me from accepting the paper at this stage. They suggest, however, that a revised version might prove acceptable, if you can address their concerns. If you think that you can deal satisfactorily with the criticisms on revision, I would be pleased to see a revised manuscript. We would then return it to the reviewers.

Please ensure that you clearly highlight all changes made in the revised manuscript. Please avoid using 'Tracked changes' in Word files as these are lost in PDF conversion.

I should be grateful if you would also provide a point-by-point response detailing how you have dealt with the points raised by the reviewers in the 'Response to Reviewers' box. Please attend to all of the reviewers' comments. If you do not agree with any of their criticisms or suggestions please explain clearly why this is so.

Reviewer 1

Advance summary and potential significance to field

Shafraz et al. generated a light activated split TurboID proximity labeling system, which theoretically allows for temporal and spatial control of proximity labeling. In their manuscript, they demonstrated the system works as designed for a known target, and showed improved signal to

© 2023. Published by The Company of Biologists under the terms of the Creative Commons Attribution License (https://creativecommons.org/licenses/by/4.0/).

noise ratio over TurboID. This work adds to a growing list of PL tools, including light-activated PL, and is well suited for publication in JCS, after minor corrections.

Comments for the author

Minor comments:

1. A similar work was made available as a preprint by the Ting lab (https://www.ncbi.nlm.nih.gov/pmc/articles/PMC10028978/) and should be cited.

2. “exposure to blue light, CRY2 and CIB1 dimerize within 300 ms (Kennedy et al., 2010; Taslimi et 90 al., 2016) and reconstitute the two halves of the split-TurboID enzyme” - The authors should not use data generated in another system to claim these are the dimerization times in their system, which differs for example by the addition of specific fusion proteins.

3. “Next, we measured the level of CibN, CryC, and Sta on the membrane; fluorescence intensities were normalized to CibN fluorescence intensity to account for differences in CibN expression levels in different cells (Fig. 2h table S1).” - It is unclear what causes the difference in CibN fluorescence intensity, as these cells differ mostly by light exposure and biotin (with the exception of 2G). The rationale of normalizing another fluorescent protein (CryC) by the expression of CibN is also unclear. At the very least unnormalized data should be made available as a supplementary.

4. “This data demonstrated that Ecad-LAB had positive hits and only 3 negative hits” - The authors should specify the source used as ground truth for Ecad interactors, and specify not only the hits, but what fraction of known interactors were observed.

5. The authors state that “It is important to note that while every Ecad-Turbo protein is capable of biotinylating a target, Ecad-LAB has a Pearson’s correlation coefficient of 0.44 (see Fig. 3), implying that only ~44% of ECibN proteins are bound to CryC and capable of biotinylating” - A Pearson correlation of 0.44 does not directly indicate that 44% of ECibN proteins are bound to CryC. Additionally, the bound fraction does not directly indicate the fraction capable of biotinylating.

6. In fig S16, the marked band is very weak in the L- 1h experiment, but strong in 3/5/18h L-, despite basically identical experimental conditions.
Additionally, the 18h L+ looks identical to L-, suggesting no labeling. These results need to be addressed in the text.

<u>Reviewer 2</u>

Advance summary and potential significance to field

This manuscript from Shafraz, Davis, and Sivasankar describes their efforts to build and test a light-activated version of split TurboID for proximity labeling. TurboID is an extremely fast biotin ligase (<10 minutes, compare to 16-24 hours for BioID or BioID2) created by the Ting lab. Though fast, it suffers from high background. To address this, the Sivasankar lab sought to create a version of TurboID that could activated on demand.

Their strategy was to fuse the two halves of split TurboID to CRY2 and CIB1, an established pair of proteins that dimerize upon exposure to blue light, a strategy they call Light Activated BioID (LAB). They showed that the LAB system works in both HEK and MDCK cells. Exposure to blue light induced dimerization and promoted biotinylation at the membrane (when CIB1 was fused to a membrane-targeted EGFP in HEK cells) or cell-cell contacts (when CIB1 was fused to the cytoplasmic tail of E-cadherin in MDCK cells). They then compared the E-cadherin interactomes identified by mass spec from MDCK cells using LAB and full-length TurboID. E-cadherin associated proteins were enriched upon exposure to blue light in E-cadherin-LAB system, validating the system. They also showed that the level of background (false positives) was lower in the LAB system versus E-cadherin-TurboID.

© 2023. Published by The Company of Biologists under the terms of the Creative Commons Attribution License (https://creativecommons.org/licenses/by/4.0/).

Overall, this study describes a new and useful proximity labeling tool - LAB - that should interest researchers using proximity proteomics. That said, they are not the first to describe a light-activated TurboID system. They cite one such study in the Discussion, work from Chen and colleagues (2022), though there are notable differences between the studies. The Ting lab also recently published their own light-activated system, LOV-Turbo (Lee et al., 2023). LOV-Turbo is a fusion between the light-activated LOV domain and TurboID that reduces background. LOV-Turbo is simpler than LAB (one construct versus two), but I feel the use of split TurboID in the LAB system could be advantageous in some situations.
Thus, even though this study is not the first to demonstrate a light-inducible proximity labeling system, I do think it describe a tool of significant interest to the larger proteomic community.

Comments for the author

The manuscript is logical and concise. The conclusions are largely supported by the data. Nonetheless, I do believe the manuscript would benefit from some reorganization and more detail, particularly when describing/discussion the mass spec results.

Major concerns/suggestions:
1. The Results section ends with a whimper - the last section (two pages) on benchmarking the biotinylation efficiency describes only supplemental data. In fact, the last figure reference (Fig. 4E) is on line 219 and the Results section ends on line 278. I think much/all of the data analyzing and comparing the biotinylation efficiency of LAB could come earlier in the manuscript, certainly before the mass spec data (Fig 4) and possibly earlier. Also, some of the current supplemental data is noteworthy (e.g., Fig S16) and could be added to an existing figure or used to create a new one.

2. The E-cadherin mass spec data comparing LAB to TurboID is interesting, but I found the analysis and Figure 4 both a little confusing. The volcano plots (4a and 4b) are a standard way to plot p-value versus fold change for mass spec hits but the use of "positive" and "negative" terms and how they are applied is confusing (or perhaps I'm missing something).
For example, I assume Fig 4a the positive candidates include identified proteins that were enriched in E-cadherin LAB expressing MDCK cells incubated with biotin and exposed to blue light versus E-cadherin LAB expressing MDCK cells incubated with biotin and kept in the dark (the control). Why then is Log2 fold change negative for these candidates and positive for the negative candidates? The same logic applies to 4b.

3. The E-cadherin interactome mass spec data in Fig 4 should be compared to existing E-cadherin BioID data sets (Itallie et al., Guo et al.). This is admittedly an extension of the study, but one that I feel is important since it would help to 1) further establish the utility of the LAB system, 2) determine how LAB and TurboID compare to BioID in MDCK cells (Itallie et al.), and 3) add important depth to the study as I expect the analysis will interest many in the adhesion field. At a minimum, the mass spec data should be collated and organized into an Excel spreadsheet and added as supplemental data.

Minor concerns/suggestions:
- Changes in the mCherry/Cry2 membrane localization shown in Fig 1 should be plotted over 600 seconds, not just 3 seconds as shown in 1E.

- The categories in Fig 4E should be in the figure, not the legend.

- In general, the labeling in the figures could be clearer, especially the supplemental figures. For example, the western blot is S16a is hard to decipher even with the legend.

- A Pearson's correlation coefficient of 0.44 does not imply that 44% of protein A overlaps with protein B (251-252).

- How many cells were used in each mass spec replicate?

© 2023. Published by The Company of Biologists under the terms of the Creative Commons Attribution License (https://creativecommons.org/licenses/by/4.0/).

- In the fluorescence intensity measurements in Fig 2h and 3h, did you measure total fluorescence (across the field of cells) or fluorescence at the membrane/cell-cell contacts?

First revision

Author response to reviewers' comments

We are very thankful to the reviewers for their valuable suggestions which have helped us improve our manuscript. We have uploaded a pdf file containing a point-by-point response to the reviewer comments in the supplementary information.

Response to Reviewers

We thank the reviewers for their valuable suggestions and are grateful to the editor for giving us this opportunity to address the reviewer comments. Our detailed responses to the reviewers' comments are listed below. The original comments are included verbatim in *italicized text*.

Reviewer # 1 Comments for the Author:

Minor comments:

1. *A similar work was made available as a preprint by the Ting lab (https://www.ncbi.nlm.nih.gov/pmc/articles/PMC10028978/) and should be cited.*

On **lines 334-341** we now describe LOV-Turbo, the technique developed by the Ting Lab (Lee et al., 2023). We state that while LOV-Turbo is simpler to use (since it requires transfection with only one construct), its ability to accurately and precisely determine the interactome of a bait protein has not been comprehensively demonstrated. This is because Lee et al. primarily used LOV- Turbo for compartmental proximity labeling and not for determining specific protein interactions with a bait. Consequently, LOV-Turbo may be preferable for cellular compartment-based searches, such as identifying proteins that traffic between specific cell compartments, while LAB is intended for proteome searches centered around a single bait protein of interest.

2. "exposure to blue light, CRY2 and CIB1 dimerize within 300 ms (Kennedy et al., 2010; Taslimi et 90 al., 2016) and reconstitute the two halves of the split-TurboID enzyme" - The authors should not use data generated in another system to claim these are the dimerization times in their system, which differs for example by the addition of specific fusion proteins.

We thank the reviewer for this comment and have clarified this important point on **lines 112-113** and on **lines 118-121** of our manuscript. In Fig. 1B, we show that upon light exposure CryC begins binding to CibN in under a second and remains bound for ~ 10 mins. Consequently, our data shows that while the interaction kinetics of our construct may differ slightly from previously reported values for CRY2/CIB1 due to the use of fusion proteins, CryC and CibN associate and dissociate at largely similar rates.

3. "Next, we measured the level of CibN, CryC, and Sta on the membrane; fluorescence intensities were normalized to CibN fluorescence intensity to account for differences in CibN expression levels in different cells (Fig. 2h, table S1)." - It is unclear what causes the difference in CibN fluorescence intensity, as these cells differ mostly by light exposure and biotin (with the exception of 2G). The rationale of normalizing another fluorescent protein (CryC) by the expression of CibN is also unclear. At the very least, unnormalized data should be made available as a supplementary.

On **lines 129-135**, we now clarify that fluorescence intensities were normalized to CibN

© 2023. Published by The Company of Biologists under the terms of the Creative Commons Attribution License (https://creativecommons.org/licenses/by/4.0/).

fluorescence intensity to account for differences in CibN expression levels in different cells. Since CibN is membrane bound while CryC is expressed in the cytoplasm, there was always significantly more CryC in a cell than CibN. The higher levels of CryC compared to CibN can be rationalized geometrically since CibN is membrane bound, it is present in quantities proportional to the cell surface area while CryC is present in quantities proportional to the cell volume. Thus, the copy number of CryC would need to be an order of magnitude less than CibN in order to fail to saturate all CibN binding sites.

As suggested by the reviewer, **we now include the unnormalized fluorescence data in supporting information (Fig. S1)**. While both the normalized and unnormalized data look similar, we believe that the normalized data allows a more accurate comparison across different cells due to differences in CibN, ECibN, and CryC expression levels. We have therefore retained the normalized data in Fig. 2H & Fig. 3H.

4. "This data demonstrated that Ecad-LAB had positive hits and only 3 negative hits" - The authors should specify the source used as ground truth for Ecad interactors, and specify not only the hits, but what fraction of known interactors were observed.

We thank the reviewer for this suggestion and have included the full list of protein hits from our MS data in the SI. Motivated by the reviewer's comments, on **lines 237-238** we clarify that the "negative hits" correspond to proteins that have higher levels in the negative conditions (-L for Ecad-LAB and -B for Ecad-Turbo), thereby throwing doubt on the legitimacy of their presence in the positive conditions.

Our hits correlate with previously published Ecad interactomes, including TurboID hits previously published in Shafraz et al. 2020. Unfortunately, it is difficult to determine the percentage of "true interactors," as that has not been fully determined for Ecad. However, all proteins previously confirmed to interact with Ecad via AFM binding assay in Shafraz et al. 2020 were found (Fig. 4A, B).

5. The authors state that "It is important to note that while every Ecad-Turbo protein is capable of biotinylating a target, Ecad-LAB has a Pearson's correlation coefficient of 0.44 (see Fig. 3), implying that only ~44% of ECibN proteins are bound to CryC and capable of biotinylating" - A Pearson correlation of 0.44 does not directly indicate that 44% of ECibN proteins are bound to CryC. Additionally, the bound fraction does not directly indicate the fraction capable of biotinylating.

We thank the reviewer for pointing this out to us. On **lines 213-215**, we now clarify that while every Ecad-Turbo protein is capable of biotinylating a target, Ecad-LAB has a Pearson's correlation coefficient of 0.44 implying that a much smaller fraction of ECibN is bound to CryC and capable of biotinylating. On **lines 148-155** and on **lines 192-195**, we also use the measured Pearson's Coefficients for CibN/CryC and for ECibN/CryC in the L+ and L- condition to calculate the coefficients of determination (the squares of the Pearson's Coefficients). These coefficients of determination indicate the fraction of CryC localization to the membrane upon light exposure which can be attributed to the presence of CibN or ECibN.

6. In fig S16, the marked band is very weak in the L- 1h experiment, but strong in 3/5/18h L-, despite basically identical experimental conditions. Additionally, the 18h L+ looks identical to L-, suggesting no labeling. These results need to be addressed in the text.

We thank the reviewer for this comment. On **lines 307-314** we now clarify that the differences in intensities between biotinylated protein bands in the L+ and L- conditions decreases as the light exposure is increased beyond 1 hour. Consequently, after 18h light exposure, the intensities of the L+ and L- bands are similar. We believe that this occurs because long periods of light exposure damage the cells and brings the harvested biotinylated protein closer to the background levels. Therefore, for longer-term experiments, the optimal light intensity and exposure cycle will likely need to be determined and further optimized based on the cells and application.

© 2023. Published by The Company of Biologists under the terms of the Creative Commons Attribution License (https://creativecommons.org/licenses/by/4.0/).

Reviewer # 2 Comments for the Author:

The manuscript is logical and concise. The conclusions are largely supported by the data. Nonetheless, I do believe the manuscript would benefit from some reorganization and more detail, particularly when describing/discussion the mass spec results.

We thank the reviewer for their suggestions and have reorganized the manuscript and clarified the results section of the manuscript.

Major concerns/suggestions:

1. The Results section ends with a whimper - the last section (two pages) on benchmarking the biotinylation efficiency describes only supplemental data. In fact, the last figure reference (Fig. 4E) is on line 219 and the Results section ends on line 278. I think much/all of the data analyzing and comparing the biotinylation efficiency of LAB could come earlier in the manuscript, certainly before the mass spec data (Fig 4) and possibly earlier. Also, some of the current supplemental data is noteworthy (e.g., Fig S16) and could be added to an existing figure or used to create a new one.

We thank the reviewer for these excellent suggestions and have reorganized the results section to better highlight our MS results. The section on benchmarking biotinylation efficiency has been moved to **lines 200-220** (before the mass spec data). The text in this section has also been extensively reduced. The mass spec section is now the last section of the results, and we think that has made the results 'pop'.

2. The E-cadherin mass spec data comparing LAB to TurboID is interesting, but I found the analysis and Figure 4 both a little confusing. The volcano plots (4a and 4b) are a standard way to plot p-value versus fold change for mass spec hits, but the use of "positive" and "negative" terms and how they are applied is confusing (or perhaps I'm missing something).

For example, I assume Fig 4a the positive candidates include identified proteins that were enriched in E-cadherin LAB expressing MDCK cells incubated with biotin and exposed to blue light versus E-cadherin LAB expressing MDCK cells incubated with biotin and kept in the dark (the control). Why then is Log2 fold change negative for these candidates and positive for the negative candidates? The same logic applies to 4b.

From the reviewer's comments, we can see that the previous volcano plots were confusing (because the x-axis was plotted in a 'less widely used' format). Previously we had plotted the x-axis as the Log2 fold change of the dark condition divided by the light condition (for Ecad-LAB) and the Log2 fold change of the -Biotin condition divided by the +Biotin condition (for Ecad-Turbo). Consequently, the positive data points were placed in the left quadrant and the negative data points in the right quadrant. Based on the reviewer's feedback, we have replotted the x-axis of the volcano plots in **Figures 4A and 4B** in a more conventional form. The x-axis of the Ecad-LAB volcano plots are plotted as the Log2 fold change of the light condition divided by the dark condition. Similarly, the x-axis of the Ecad-Turbo volcano plots are plotted as the Log2 fold change of the +Biotin condition divided by the -Biotin condition. Consequently, the positive data points are now in the right quadrant while the negative data points in the left quadrant.

On **lines 237-238** we clarify that the "negative hits" correspond to proteins that have higher levels in the negative conditions (absence of light for Ecad-LAB and absence of biotin for Ecad-Turbo), thereby throwing doubt on the legitimacy of their presence in the positive conditions. Our data shows that LAB has far fewer of these false positives (3 for LAB *vs.* 139 for Ecad-Turbo), indicating less background labelling in LAB.

3. The E-cadherin interactome mass spec data in Fig 4 should be compared to existing E-cadherin BioID data sets (Itallie et al., Guo et al.). This is admittedly an extension of the study, but one that I feel is important since it would help to 1) further establish the utility of the LAB system, 2) determine how LAB and TurboID compare to BioID in MDCK cells (Itallie et al.), and 3) add important depth to the study as I expect the analysis will interest many in the adhesion field. At a minimum, the mass spec data should be collated and organized into an Excel spreadsheet

© 2023. Published by The Company of Biologists under the terms of the Creative Commons Attribution License (https://creativecommons.org/licenses/by/4.0/).

and added as supplemental data.

As suggested by the reviewer, we have now included a collated list of hits for both Ecad-LAB and Ecad-Turbo as an **Excel spreadsheet in the supplemental data**. Since our manuscript already benchmarks LAB against TurboID, the 'gold-standard' proximity labeling approach in the field, we respectfully believe that our analysis is sufficient to establish the accuracy and precision of LAB. Since the goal of our manuscript is to present LAB as a resource for the cell biology community, we respectfully believe that a more detailed analysis of the E-cadherin interactome is beyond the scope of this manuscript.

Minor concerns/suggestions:

- Changes in the mCherry/Cry2 membrane localization shown in Fig 1 should be plotted over 600 seconds, not just 3 seconds as shown in 1E.

We thank the reviewer for their suggestion and have added an additional graph to Figure 1 (**Fig. 1D**) that shows the CryC membrane intensity over the 600+ seconds.

- The categories in Fig 4E should be in the figure, not the legend.

We thank reviewer for their suggestion. However, when we tried incorporating this change, we realized that adding categories to the figure made it unwieldy and difficult to read. So, we respectfully have retained the original labeling scheme.

- In general, the labeling in the figures could be clearer, especially the supplemental figures. For example, the western blot is S16a is hard to decipher even with the legend.

We have now increased the clarity of the figures' labeling in accordance with the reviewer's suggestion.

- A Pearson's correlation coefficient of 0.44 does not imply that 44% of protein A overlaps with protein B (251-252).

We thank the reviewer for this comment and have changed the manuscript so it no longer suggests that the Pearson's coefficient directly implies percentage of protein overlap. (See answer to Reviewer 1's 5th comment).

- How many cells were used in each mass spec replicate?

On **lines 442-443** of the methods section, we now state that approximately 8 x 10^7 cells were used for each replicate.

- In the fluorescence intensity measurements in Fig 2h and 3h, did you measure total fluorescence (across the field of cells) or fluorescence at the membrane/cell-cell contacts?

We have updated the manuscript (**lines 129, 169, 550, 575**) to clarify that fluorescence was measured at the cell membranes, with ROI's chosen using the Cib fluorescence as a membrane indicator.

Second decision letter

MS ID#: JOCES/2023/261430

MS TITLE: Light Activated BioID (LAB): an optically activated proximity labeling system to study protein-protein interactions.

AUTHORS: Omer Shafraz, Carolyn Marie Orduno Davis, and Sanjeevi Sivasankar

© 2023. Published by The Company of Biologists under the terms of the Creative Commons Attribution License (https://creativecommons.org/licenses/by/4.0/).

ARTICLE TYPE: Tools and Resources

I am happy to tell you that your manuscript has been accepted for publication in Journal of Cell Science, pending standard ethics checks.

Reviewer 1

Advance summary and potential significance to field

The authors fully addressed all points raised. This manuscript will be a welcomed addition to the field of proximity labeling.

Comments for the author

.

Reviewer 2

Advance summary and potential significance to field

This manuscript from Shafraz, Davis, and Sivasankar describes a new and useful proximity labeling tool - LAB - that should interest researchers using proximity proteomics. Their strategy fuses the two halves of split TurboID to CRY2 and CIB1, an established pair of proteins that dimerize upon exposure to blue light, which they call Light Activated BioID (LAB).
They showed that the LAB system works in both HEK and MDCK cells. Exposure to blue light induces dimerization and promoted biotinylation at the membrane (when CIB1 was fused to a membrane-targeted EGFP in HEK cells) or cell-cell contacts (when CIB1 was fused to the cytoplasmic tail of E-cadherin in MDCK cells). E-cadherin associated proteins were enriched upon exposure to blue light in E-cadherin-LAB system, validating the system. The level of background (false positives) was lower in the LAB system versus E-cadherin-TurboID.

Comments for the author

The authors have addressed my concerns. Well done.

© 2023. Published by The Company of Biologists under the terms of the Creative Commons Attribution License (https://creativecommons.org/licenses/by/4.0/).
